# A Classic Near-Infrared Probe Indocyanine Green for Detecting Singlet Oxygen

**DOI:** 10.3390/ijms17020219

**Published:** 2016-02-06

**Authors:** Cheng-Yi Tang, Feng-Yao Wu, Min-Kai Yang, Yu-Min Guo, Gui-Hua Lu, Yong-Hua Yang

**Affiliations:** 1 State Key Laboratory of Pharmaceutical Biotechnology, NJU-NJFU Joint Institute of Plant Molecular Biology, School of Life Sciences, Nanjing University, Nanjing 210093, China; tangchengyi_nju@163.com (C.-Y.T.); cdwfyao@126.com (F.-Y.W.); 13404159963@163.com (M.-K.Y.); 15856342898@163.com (Y.-M.G.); guihua.lu@nju.edu.cn (G.-H.L.); 2 Co-Innovation Center for Sustainable Forestry in Southern China, Nanjing Forestry University, Nanjing 210037, China

**Keywords:** singlet oxygen (^1^O_2_), indocyanine green (ICG)

## Abstract

The revelation of mechanisms of photodynamic therapy (PDT) at the cellular level as well as singlet oxygen (^1^O_2_) as a second messengers requires the quantification of intracellular ^1^O_2_. To detect singlet oxygen, directly measuring the phosphorescence emitted from ^1^O_2_ at 1270 nm is simple but limited for the low quantum yield and intrinsic efficiency of ^1^O_2_ emission. Another method is chemically trapping ^1^O_2_ and measuring fluorescence, absorption and Electron Spin Resonance (ESR). In this paper, we used indocyanine green (ICG), the only near-infrared (NIR) probe approved by the Food and Drug Administration (FDA), to detect ^1^O_2_ *in vitro*. Once it reacts with ^1^O_2_, ICG is decomposed and its UV absorption at 780 nm decreases with the laser irradiation. Our data demonstrated that ICG could be more sensitive and accurate than Singlet Oxygen Sensor Green reagent^®^ (SOSG, a commercialized fluorescence probe) *in vitro*, moreover, ICG functioned with Eosin Y while SOSG failed. Thus, ICG would reasonably provide the possibility to sense ^1^O_2_ *in vitro*, with high sensitivity, selectivity and suitability to most photosensitizers.

## 1. Introduction

Singlet oxygen (^1^O_2_), a highly reactive oxygen species (ROS), plays a crucial role in photodynamic therapy (PDT) by causing oxidative damage to proteins, DNA and lipids [1,2,3]. In addition, ^1^O_2_ is proposed to be a second messenger in cell signaling transduction [4]. Nevertheless, the detailed molecular mechanisms of PDT at the cellular level as well as ^1^O_2_ as a second messenger are not fully understood yet, partly due to limitations of quantification of intracellular ^1^O_2_ such as short half-life and high reactivity. Therefore, development of high sensitivity methods for detecting ^1^O_2_ *in vivo* is an appealing challenge [5,6,7,8].

Directly measuring the light emission at *ca*. 1270 nm of ^1^O_2_ is frequently used for ^1^O_2_ detection and characterization, but this method is limited for the low quantum yield (*ca.* 10^−7^) [9] and the intrinsic low efficiency of singlet-oxygen emission, especially in physiological environments where the lifetime of ^1^O_2_ is fairly short (3.1 μs) [10]. Chemical trapping of ^1^O_2_ is also applied extensively, and the detecting methods include fluorescence, absorption and Electron Spin Resonance (ESR). Fluorescence probes for ^1^O_2_ have drawn much attention, including DPAX or DMAX [11], ATTA-Eu^3+^ [12] and Singlet Oxygen Sensor Green reagent^®^ (SOSG). They use an anthracene moiety to trap ^1^O_2_ that quenches the fluorescence of the fluorophore through an electron transfer reaction. Once it trapps ^1^O_2_, the resultant oxygen adduct fails to be a functional intramolecular electron donor, and the fluorescence is recovered. For example, SOSG emits nattier blue fluorescence at 395 and 416 nm, under excitation at 372 and 393 nm. Upon reaction with ^1^O_2_, the immediate product SOSG endoperoxide (SOSG-EP) exhibits green fluorescence, with excitation and emission peak at 504 and 525 nm, respectively [13]. These probes are convenient, highly sensitive and widely used, especially SOSG, which has been broadly used in recent studies [14,15,16,17]. However, fluorescence probes have several drawbacks. One is the lack of specificity when used with some photosensitizers. Many ^1^O_2_ sensitizers have a pronounced absorption band in the visible spectrum. If there is overlap with the emission peak of fluorophores of the probe, the signal produced by reaction with ^1^O_2_ will be diminished or even eliminated. False-negative signals arise in these situations to camouflage the signal enhancement evoked by ^1^O_2_. For instance, xanthene dyes such as rose bengal and eosin Y exhibit intense absorption bands in 525 nm, which happens to be the emission peak wavelength of SOSG-EP [18]. In this paper, we clarified the interference of eosin Y on SOSG to detect ^1^O_2_. Moreover, compared with ESR and absorption measurement, fluorescence signals are more unstable and temporal to detect.

In consideration of remedying these defects, we focused on developing new ^1^O_2_ probes. Serendipitously, we discovered for the first time that UV absorption of indocyanine green (ICG) at 780 nm decreased under 660 nm laser irradiation in chlorin e6 (Ce6) solution. As another tricarbocyanine, ICG is the only near-infrared (NIR) probe approved by the Food and Drug Administration (FDA) and has been used in clinical therapy for over 30 years with a high safety record [19]. ICG is a negatively charged and amphiphilic tricarbocyanine, with an absorption peak at ~807  nm and an emission peak at ~822  nm [20]. In this paper, we conducted systematic experiments to substantiate that ICG can function as an ^1^O_2_ probe. Once irradiated under 660 nm laser light, Ce6 can produce ^1^O_2_, which decomposes ICG through the break of alkene (double bond in a polymethine chain) hypothetically (Supplementary Material, Figure S1) [21]. This probe is safe, sensitive and broadly suited for most photosensitizers. To better evaluate its detecting ability, we chose the commercialized ^1^O_2_ probe SOSG as a reference probe.

## 2. Results and Discussion

**Figure 1:**
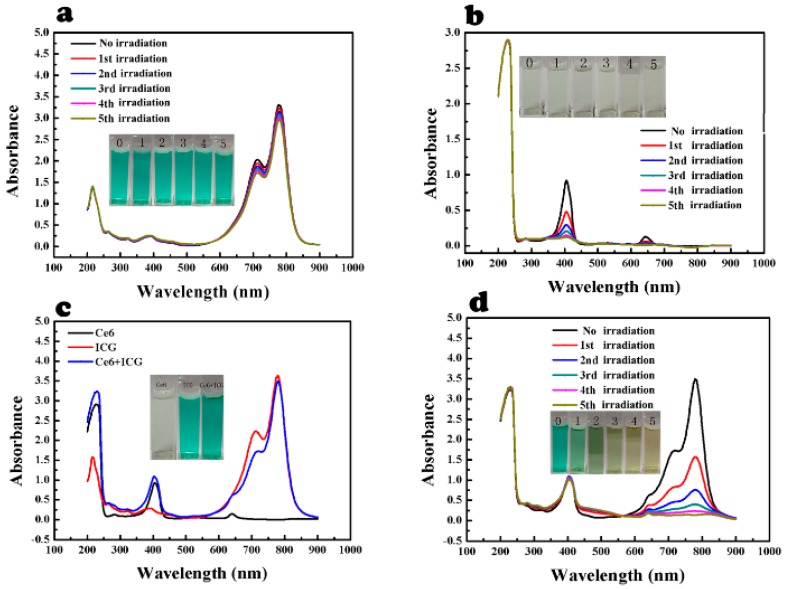
Feasibility of ICG to detect ^1^O_2_. (**a**) ICG under laser irradiation; (**b**) Ce6 under laser irradiation; (**c**) ICG, Ce6 and ICG with Ce6; (**d**) ICG with Ce6 under laser irradiation.

The feasibility of ICG to detect ^1^O_2_ produced by Ce6 was assessed (Figure 1). The intensity of maximum absorption (at 780 nm) of ICG solution (25 μg/mL) slightly decreased after each laser irradiation (Figure 1a). This indicated that ICG was sufficiently stable under 660 nm laser irradiation in the medium. In contrast, absorption of Ce6 (5 μg/mL) at 400 and 650 nm significantly reduced under laser irradiation as expected (Figure 1b). This indicated that Ce6 completely decomposed after 15 min laser irradiation. The UV absorption spectra of mixed solution of ICG and Ce6 was also examined with and without laser irradiation, respectively (Figure 1c,d). Ce6 had no influence on absorption of ICG at 780 nm (Figure 1c). The solution was exposed to laser irradiation, then the absorption of ICG at 780 nm significantly reduced after each instance of laser irradiation (Figure 1d). The decrease of UV absorption was accordant with the visible bleaching of the green color of ICG as the picture denoted (Figure 1d), which was attributed to the decomposition of ICG by ^1^O_2_ generated by Ce6. The absorption decline of ICG was correlated positively with the time of laser irradiation, which can be explained by the increment of amount produced by Ce6 under continuous laser irradiation [22]. Therefore, we proposed that it was feasible to use ICG as a singlet oxygen probe.

**Figure 2:**
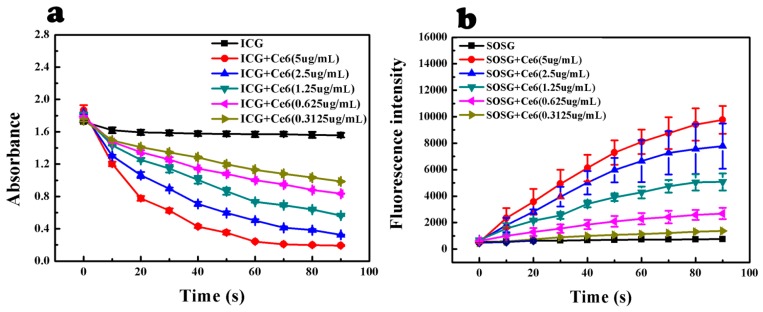
Sensitivity of ICG to ^1^O_2_ produced by Ce6. (**a**) UV absorbance of ICG with Ce6 after each 10-s laser irradiation (nine times in total); (**b**) fluorescence intensity of SOSG with Ce6 after each 10-s laser irradiation (nine times in total). (Mean ± SD, *n* = 3)

The sensitivity of ICG towards ^1^O_2_ was assessed by using ICG to detect different concentrations of ^1^O_2_ (Figure 2 and Table S1 (Supplementary Material)). The ^1^O_2_ concentration was proportional to Ce6 concentration and laser irradiation time [22]. Both of the decreases in UV absorption of ICG and increase in fluorescence intensity of SOSG displayed a concentration-dependent manner, which further confirmed that ICG could function as an ^1^O_2_ probe. There were significant differences of ICG absorption intensity between each adjacent Ce6 concentration group (*p* < 0.05); however, SOSG failed to distinguish high Ce6 concentration groups (2.5 and 5 μg/mL) (*p* > 0.05). Therefore, it was assumed that ICG was more accurate than SOSG.

**Figure 3:**
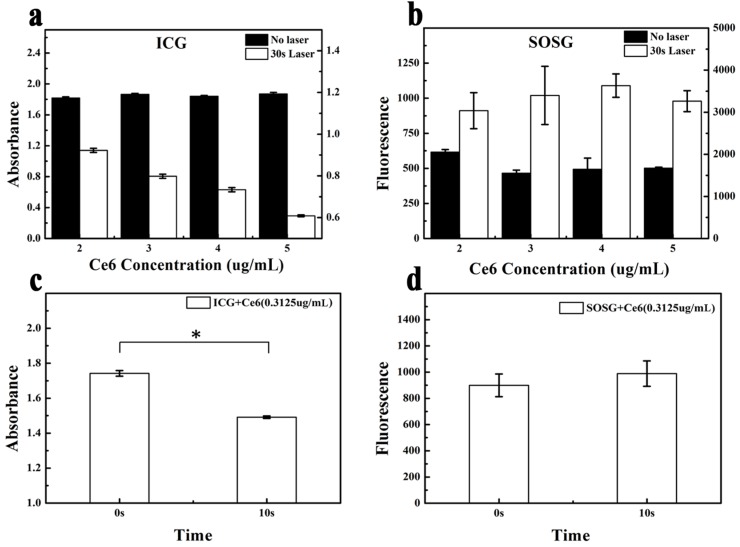
Accuracy of ICG to detect ^1^O_2_. (**a**) UV absorbance of ICG with different Ce6 concentrations after 30 s laser irradiation (*, *p* < 0.05); (**b**) fluorescence intensity of SOSG with different Ce6 concentrations after 30 s laser irradiation; (**c**) absorbance of ICG with the lowest Ce6 concentration after 10 s laser irradiation (*, *p* < 0.05); (**d**) fluorescence intensity of SOSG with the lowest Ce6 concentration after 10 s of laser irradiation. (Mean ± SD, *n* = 3).

To verify the high accuracy of ICG for ^1^O_2_ detection, we used ICG and SOSG to detect ^1^O_2_ produced by Ce6 at both high and low concentration with consistent laser irradiation time (Figure 3 and Table S2 (Supplementary Material)). The decrease in absorbance of ICG was correlated positively with an increase of Ce6 concentration from 2 to 5 μg/mL, as demonstrated above, and the differences among the four groups were significant (*p* < 0.05) (Figure 3a). However, there were no significant differences between the SOSG fluorescence intensity of each adjacent concentration group after laser irradiation (Figure 3b). This result confirmed the higher detecting accuracy of ICG than SOSG. In addition, it indicated that ICG has a wider detection scope than SOSG, hence we compared the detecting performance of ICG with SOSG at very low ^1^O_2_ concentration (Figure 3c,d). The mixed solutions of ICG or SOSG with Ce6 at 0.31625 μg/mL were prepared, and all the samples were under 10-s laser irradiation. The results showed that the ICG absorption decreased significantly after laser irradiation (*p* < 0.05), but there was no significant increase of fluorescence intensity of SOSG. Our data denoted that ICG could detect a wider range of ^1^O_2_ concentration and perform more accurately than SOSG.

To demonstrate that ICG could detect a broader range of photosensitizers than SOSG, we chose Eosin Y as the photosensitizer and scanned the absorption and emission spectra of mixed solutions of Eosin Y with either ICG or SOSG, respectively (Figure S2, Supplementary Material). Eosin Y had no influence on absorption of ICG at 780 nm, like Ce6 did. In contrast, it exhibited a fluorescence emission peak at 525 nm, overlapped with the detection range of SOSG.

**Figure 4:**
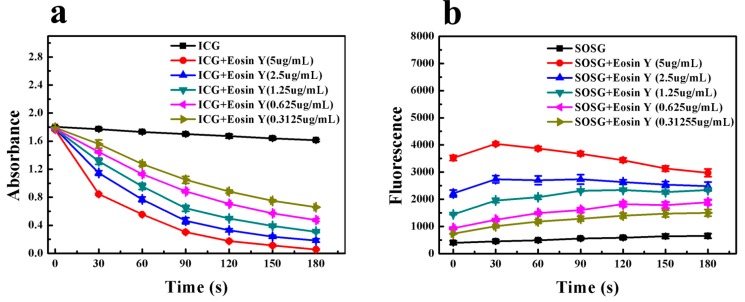
Comparison of sensitivity of ICG and SOSG on ^1^O_2_ produced by Eosin Y. (**a**) UV absorbance of ICG with Eosin Y after each 30-s 532 nm laser irradiation (six times in total); (**b**) fluorescence intensity of SOSG with Eosin Y after each 30-s laser irradiation (six times in total). (Mean ± SD, *n* = 3).

We compared the sensitivity of ICG and SOSG on ^1^O_2_ produced by Eosin Y. ICG was sufficiently stable under 532 nm laser irradiation (Figure S3a, Supplementary Material), and the absorption decrease of ICG at 780 nm with an increasing amount of ^1^O_2_ was significant (Figure S3b, Supplementary Material). The decrease in UV absorption displayed a concentration-dependent manner (Figure 4a). There were significant differences of ICG absorption intensity between each adjacent Eosin Y concentration group (*p* < 0.05). In contrast, SOSG failed to exhibit similar increments of fluorescence intensity under laser irradiation as SOSG and Ce6 mixture did (Figure 4b). This was tentatively explained by fluorescence resonance energy transfer (FRET), that is the energy emitted by SOSG-EP was absorbed by Eosin Y spontaneously, so the fluorescence measured is mostly from Eosin Y itself. One piece of evidence is the high molar extinction value of Eosin Y at 525 nm, the emission peak of SOSG (Figure S4, Supplementary Material). Moreover, the fluorescence intensity of SOSG before laser irradiation displayed an Eosin Y concentration-dependent manner. Considering the emission peak of Eosin Y at 525 nm, this fluorescence increment could be explained by the concentration-dependent increase of its emission at 525 nm (Figure S2, Supplementary Material). Another corroborative piece of evidence was the fluorescence intensity variation of Eosin Y upon 532 nm laser irradiation, which exhibited similar tendency and value to that of mixtures with SOSG (Figure S5, Supplementary Material). Finally, considering the intracellular environment is complicated and other reactive species like reactive oxygen species (ROS) and biological antioxidants like ascorbic acid (Vc) may influence the function of ICG, we examined its selectivity (Figure S6, Supplementary Material). Results showed that neither Vc nor H_2_O_2_ had a significant effect on the absorbance of ICG in the absence of ^1^O_2_, therefore, ICG possessed a high sensitivity towards ^1^O_2_.

## 3. Materials and Methods

### 3.1. Materials

UV absorption spectra of ICG were measured with UV spectrophotometer (Shimadzu, UV-2450, Kyoto, Japan). The UV absorption of ICG (at 780 nm) and the fluorescence of SOSG (λ_ex_/λ_em_ = 504/525 nm) were measured by a microplate reader. Laser irradiation was performed by two laser devices (660 nm, 1 w/cm^2^ and 532 nm, 0.1 w/cm^2^). Ce6, ICG and Eosin Y were purchased from Sigma-Aldrich Co. LLC (Shanghai, China). SOSG was purchased from Life Technologies (Shanghai, China).

### 3.2. Feasibility of ICG to Detect ^1^O_2_

Samples of ICG (25 μg/mL), Ce6 (6 μg/mL) and their mixture were prepared. The solvent is water. Absorption of each sample was measured with or without 660 nm laser irradiation. Laser irradiation was performed five times in total and 3 min each time.

### 3.3. Sensitivity of ICG to Detect ^1^O_2_

The mixed solutions of ICG (25 μg/mL) or SOSG (6 μg/mL) with different concentrations of Ce6 (0.3125, 0.625, 1.25, 2.5 and 5 μg/mL) were prepared and exposed under ten times of 660 nm laser irradiation (10 s each time). The UV absorption of ICG (at 780 nm) and the fluorescence of SOSG (λ_ex_/λ_em_ = 504/525 nm) were measured after each irradiation by a microplate Reader.

### 3.4. Accuracy of ICG to Detect ^1^O_2_

The mixed solutions of ICG (25 μg/mL) or SOSG (6 μg/mL) with four Ce6 concentrations (2, 3, 4 and 5 μg/mL) were prepared. Afterwards, the absorbance of ICG (at 780 nm) and fluorescence intensity of SOSG (λ_ex_/λ_em_ = 504/525 nm) were measured before and after 30-s 660 nm laser irradiation, respectively. The mixed solutions of ICG or SOSG with Ce6 (0.31625 μg/mL) were prepared, and all samples were under 10-s 660 nm laser irradiation before the absorbance of ICG (at 780 nm) and fluorescence intensity of SOSG (λ_ex_/λ_em_ = 504/525 nm) were measured.

### 3.5. Comparison of Sensitivity of ICG and SOSG on ^1^O_2_ Produced by Eosin Y

The water solutions of ICG (25 μg/mL) or SOSG (6 μg/mL) with five concentrations of Eosin Y (0.31255, 0.625, 1.25, 2.5 and 5 μg/mL) were prepared and exposed under six times of 532 nm laser irradiation (30 s each time). The UV absorption of ICG (at 780 nm) and the fluorescence of SOSG (λ_ex_/λ_em_ = 504/525 nm) were measured after each irradiation by a microplate reader.

### 3.6. Disturbance of Eosin Y on Absorption Spectrum of ICG

Samples of ICG (25 μg/mL), Eosin Y (2.5 μg/mL) and their mixture were prepared. UV absorption spectra of each sample were scanned. Similarly, samples of SOSG (6 μg/mL), Eosin Y (2.5 μg/mL) and their mixture were prepared. Fluorescence spectrum was scanned (λ_ex_/λ_em_ = 504/525 nm).

### 3.7. Feasibility of ICG to Detect ^1^O_2_ Produced by Eosin Y

Samples of ICG (25 μg/mL), Eosin Y (2.5 μg/mL) and their mixture were prepared. The solvent is water. Absorption of each sample was measured with or without 532 nm laser irradiation, respectively. Laser irradiation was performed five times in total and 3 min each time.

### 3.8. Tentative Explanation of Eosin Y Interference

Different concentrations of Eosin Y water solution (0.3125, 0.625, 1.25, 2.5 and 5 μg/mL) were prepared and exposed under six times of 532 nm laser irradiation (30 s each time). The fluorescence of Eosin Y (λ_ex_/λ_em_ = 504/525 nm) was measured after each irradiation.

### 3.9. The Selectivity of ICG

The mixed solutions of SOSG (6 μg/mL) and VitC (4.4 μg/mL) or H_2_O_2_ (3.4 μg/mL) with and without Ce6 (2.5 μg/mL) were prepared. The same set of solutions in which SOSG was substituted with ICG (20 μg/mL) were prepared. All of the mixed solutions without Ce6 were placed at 25 °C for 10 min before the absorbance of ICG was examined. Similarly, mixed solutions with Ce6 were irradiated by laser light for 90 s to produce ^1^O_2_ before the SOSG fluorescence and ICG absorption were measured.

## 4. Conclusions

To detect ^1^O_2_, directly measuring the phosphorescence emitted from ^1^O_2_ at 1270 nm is simple but limited for the low quantum yield and intrinsic efficiency of ^1^O_2_ emission. SOSG, a commercialized fluorescence probe, is widely used but unsuitable for photosensitizers whose absorption bands overlap the emission band of SOSG endoperoxide. In our paper, the results show that ICG, an NIR probe approved by the FDA, could be more sensitive and accurate than SOSG for ^1^O_2_ detection *in vitro*. In addition, ICG is water soluble, non-toxic and suitable for most photosensitizers. Therefore, ICG would have application prospects in detecting ^1^O_2_ *in vitro*.

## Supplementary Materials

Supplementary materials can be found at http://www.mdpi.com/1422-0067/17/2/219/s1.
