# Supplementary material for: A Classic Near-Infrared Probe Indocyanine Green for Detecting Singlet Oxygen
Source: Int J Mol Sci. 2016 Feb 6;17(2):219. doi: 10.3390/ijms17020219 (PMC4783951; doi:10.3390/ijms17020219)
Supplement: Supplementary file 1 [file ijms-17-00219-s001.pdf]

Supplementary Materials: A Classic Near-Infrared Probe Indocyanine Green for Detecting Singlet Oxygen

Cheng-Yi Tang, Feng-Yao Wu, Min-Kai Yang, Yu-Min Guo, Gui-Hua Lu and Yong-Hua Yang

Table S1. Fluorescence of SOSG and Absorption of ICG before and after 10s laser radiation.

ICG/SOSG	Before Radiation	10 s of Radiation	*p*
	Fluorescence of SOSG		
SOSG + Ce6 (0.3125 μg/mL)	984.00	1099.00	0.30
	811.00	917.00	
	903.00	950.00	
	Absorption of ICG		
ICG + Ce6 (0.3125 μg/mL)	1.76	1.49	0.00002
	1.74	1.48	
	1.73	1.50	

Table S2. Relative standard deviation (RSD) of the absorptive or fluorescent data during laser radiation.

RSD	Time of Radiation									
ICG/SOSG	0 s	10 s	20 s	30 s	40 s	50 s	60 s	70 s	80 s	90 s
ICG + Ce6 (0 μg/mL)	1.3%	1.3%	1.3%	1.6%	1.6%	1.7%	1.8%	1.9%	2.1%	2.2%
SOSG + Ce6 (0 μg/mL)	8.4%	5.8%	10.2%	10.1%	10.5%	13.2%	12.6%	12.9%	13.1%	15.0%
ICG + Ce6 (0.15625 μg/mL)	1.9%	1.9%	1.3%	1.8%	2.1%	2.6%	1.4%	2.0%	1.9%	1.9%
SOSG + Ce6 (0.15625 μg/mL)	5.9%	7.6%	7.4%	8.0%	7.5%	10.1%	8.9%	13.4%	10.5%	9.3%
ICG + Ce6 (0.3125 μg/mL)	2.8%	1.3%	0.0%	1.2%	2.3%	1.6%	1.8%	2.7%	1.6%	2.2%
SOSG + Ce6 (0.3125 μg/mL)	12.5%	6.0%	8.7%	9.6%	9.8%	9.0%	11.3%	11.5%	10.5%	8.9%
ICG + Ce6 (0.625 μg/mL)	0.7%	3.2%	3.0%	2.4%	2.3%	2.6%	2.8%	2.2%	0.6%	2.8%
SOSG + Ce6 (0.625 μg/mL)	7.8%	2.7%	23.0%	20.1%	19.0%	19.4%	18.5%	19.5%	14.5%	15.9%
ICG + Ce6 (1.25 μg/mL)	1.5%	1.7%	3.1%	2.6%	4.8%	6.4%	5.6%	5.5%	4.9%	1.8%
SOSG + Ce6 (1.25 μg/mL)	11.3%	26.1%	3.0%	10.2%	7.5%	7.4%	10.6%	10.0%	12.1%	12.7%
ICG + Ce6 (2.5 μg/mL)	1.5%	1.7%	5.6%	6.0%	4.8%	8.2%	3.6%	1.0%	1.9%	0.7%
SOSG + Ce6 (2.5 μg/mL)	9.2%	30.2%	6.0%	18.8%	17.4%	15.4%	23.8%	22.5%	24.9%	21.9%
ICG + Ce6 (5 μg/mL)	2.3%	7.1%	3.7%	6.7%	7.6%	3.4%	3.2%	2.3%	3.0%	1.4%
SOSG + Ce6 (5 μg/mL)	16.5%	32.6%	27.3%	21.7%	16.4%	12.7%	11.4%	13.7%	13.0%	10.8%

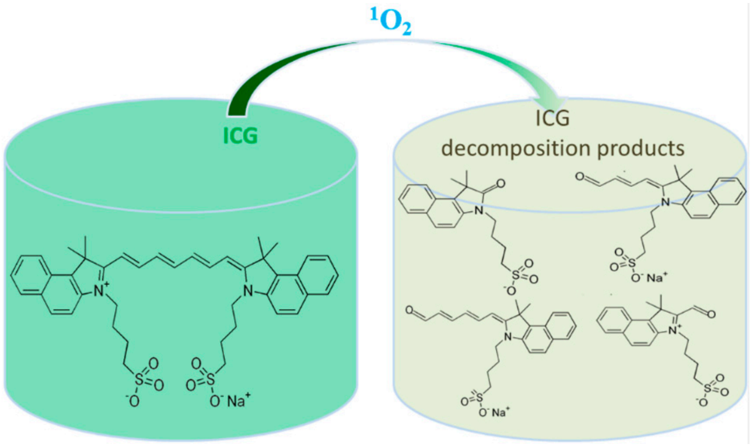

Figure S1. Tentative scheme of ICG Decomposition.

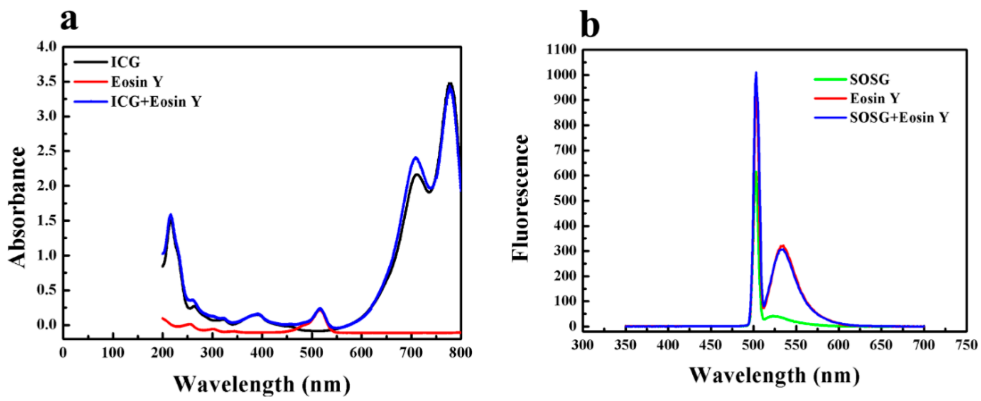

Figure S2. Disturbance of Eosin Y on absorption spectrum of ICG. (**a**) The overlay of absorbance spectra; (**b**) the overlay of fluorescence spectra ($\lambda_{ex}/\lambda_{em}$ = 504/525 nm).

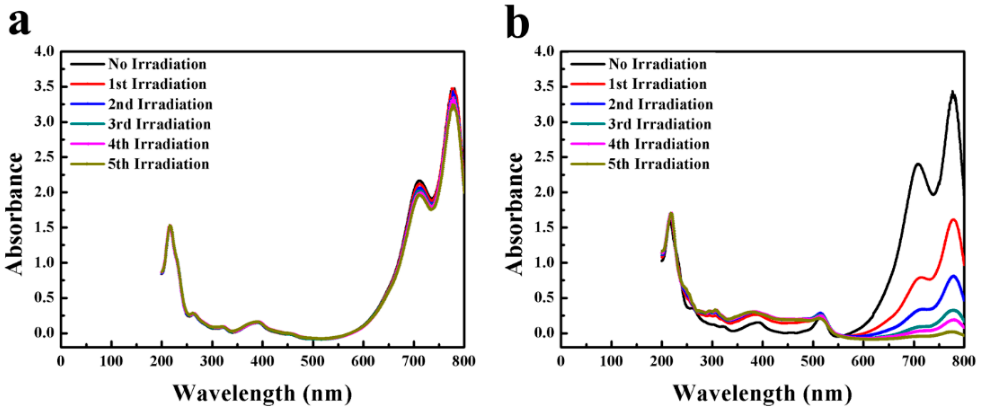

Figure S3. Feasibility of ICG to detect $^1O_2$ produced by Eosin Y. (**a**) Stability of ICG under 532 nm irradiation; (**b**) sensitivity of ICG to $^1O_2$ produced by Eosin Y.

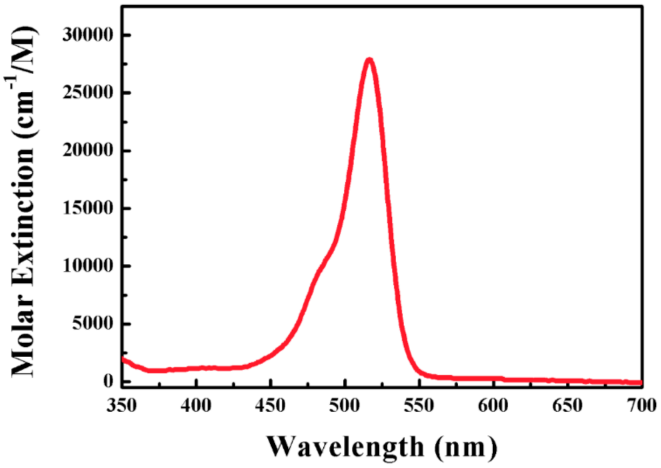

Figure S4. Absorption spectrum of Eosin Y in water.

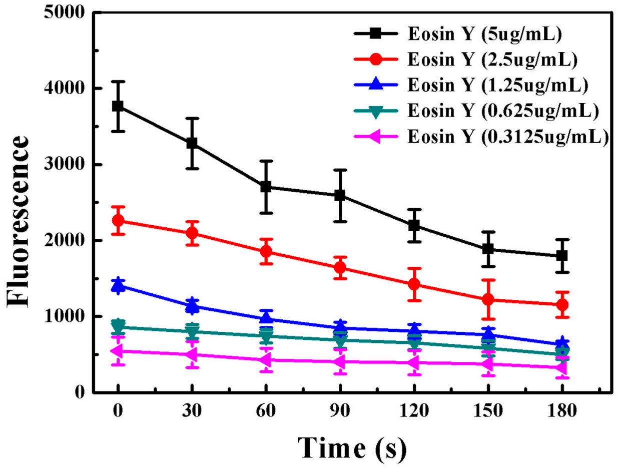

Figure S5. Tentative explanation of Eosin Y interference.

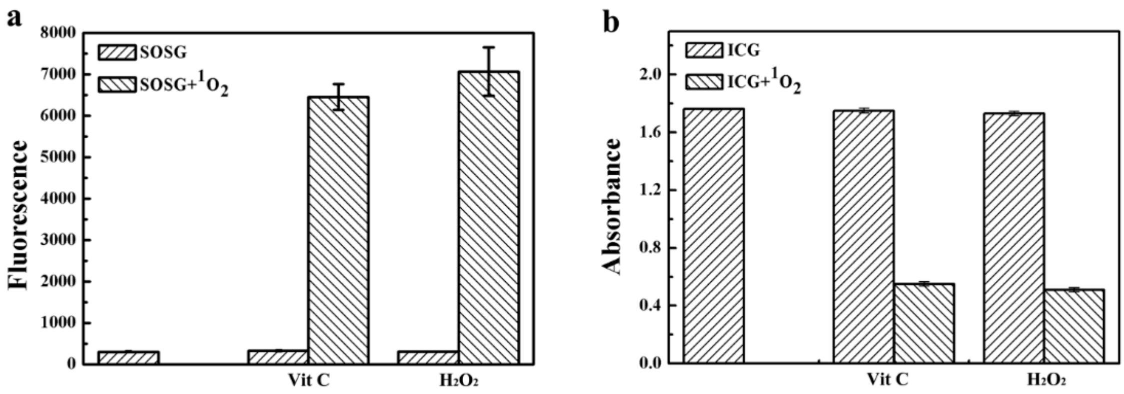

Figure S6. The selectivity of ICG. (**a**) The anti-interference ability of SOSG in detecting $^1O_2$; (**b**) the anti-interference ability of ICG in detecting $^1O_2$. (Mean ± SD, *n* = 3).
